# 870. Coverage, Cost Sharing, and Out-of-Pocket Costs for Single-Tablet HIV Antiretroviral Regimens in Qualified Health Plans in the United States, 2018-2020

**DOI:** 10.1093/ofid/ofab466.1065

**Published:** 2021-12-04

**Authors:** Rohan Khazanchi, Samuel D Powers, Amy Killelea, Kathleen A McManus

**Affiliations:** 1 College of Medicine, University of Nebraska Medical Center, Omaha, NE; 2 University of Virginia, Charlottesville, Virginia; 3 Killelea Consulting LLC, Arlington, Virginia

## Abstract

**Background:**

A key pillar of the US “Ending the HIV Epidemic” (EHE) plan is rapidly providing antiretroviral therapy (ART) to achieve viral suppression. However, access to ART is hindered by discriminatory benefit design through non-coverage, adverse tiering (including pricier cost sharing via coinsurance instead of copays), and excessive and arbitrary utilization management for ART, all of which make rapid access to HIV treatment challenging. To understand how ACA Qualified Health Plan (QHP) formularies adapt in response to new ART single tablet regimens (STRs), we analyzed QHP coverage of two first-line STRs: dolutegravir/abacavir/lamivudine (Triumeq; approved 2014) and bictegravir/emtricitabine/tenofovir alafenamide (Biktarvy; approved 2018).

**Methods:**

For all QHPs offered in the 2018-2020 ACA Marketplaces, we analyzed Biktarvy and Triumeq coverage, cost sharing, and out-of-pocket (OOP) costs at state, regional, and EHE priority jurisdiction levels.

Figure 1. Qualified Health Plan Coverage of Triumeq and Biktarvy by State, 2018-2020

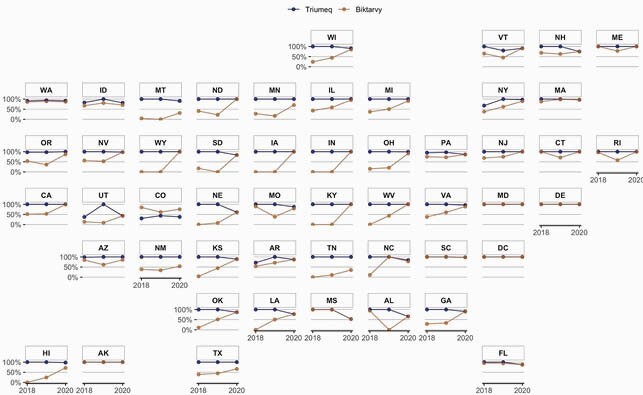

**Results:**

For 2018, 2019, and 2020, respectively, we identified 19,533, 17,007, and 21,547 QHPs. In 2018, 26 states had < 50% of QHPs covering Biktarvy, and 9 states had 0%. Conversely, 41 states had 100% of QHPs covering Triumeq, and only 2 states had < 50% (Fig. 1). Biktarvy coverage improved from 2018-2020, especially in the Midwest (27% to 88%). Improvements were driven by increased coverage with copay except in the South, where coverage with copay remained stagnant and coverage with coinsurance increased (22% to 33%) (Fig. 2). Biktarvy coverage increased in EHE jurisdictions from 74% to 90%, driven by increased coverage with coinsurance (20% to 34%) (Fig. 3). Although Biktarvy had a higher national average wholesale price than Triumeq (&4,073 vs. &3,639 per month in 2020, respectively), monthly OOP cost trends only differed regionally in the Midwest and did not differ by EHE priority jurisdiction status (Fig. 4).

Figure 2. Qualified Health Plan Coverage and Cost Sharing for Triumeq and Biktarvy by Region, 2018-2020

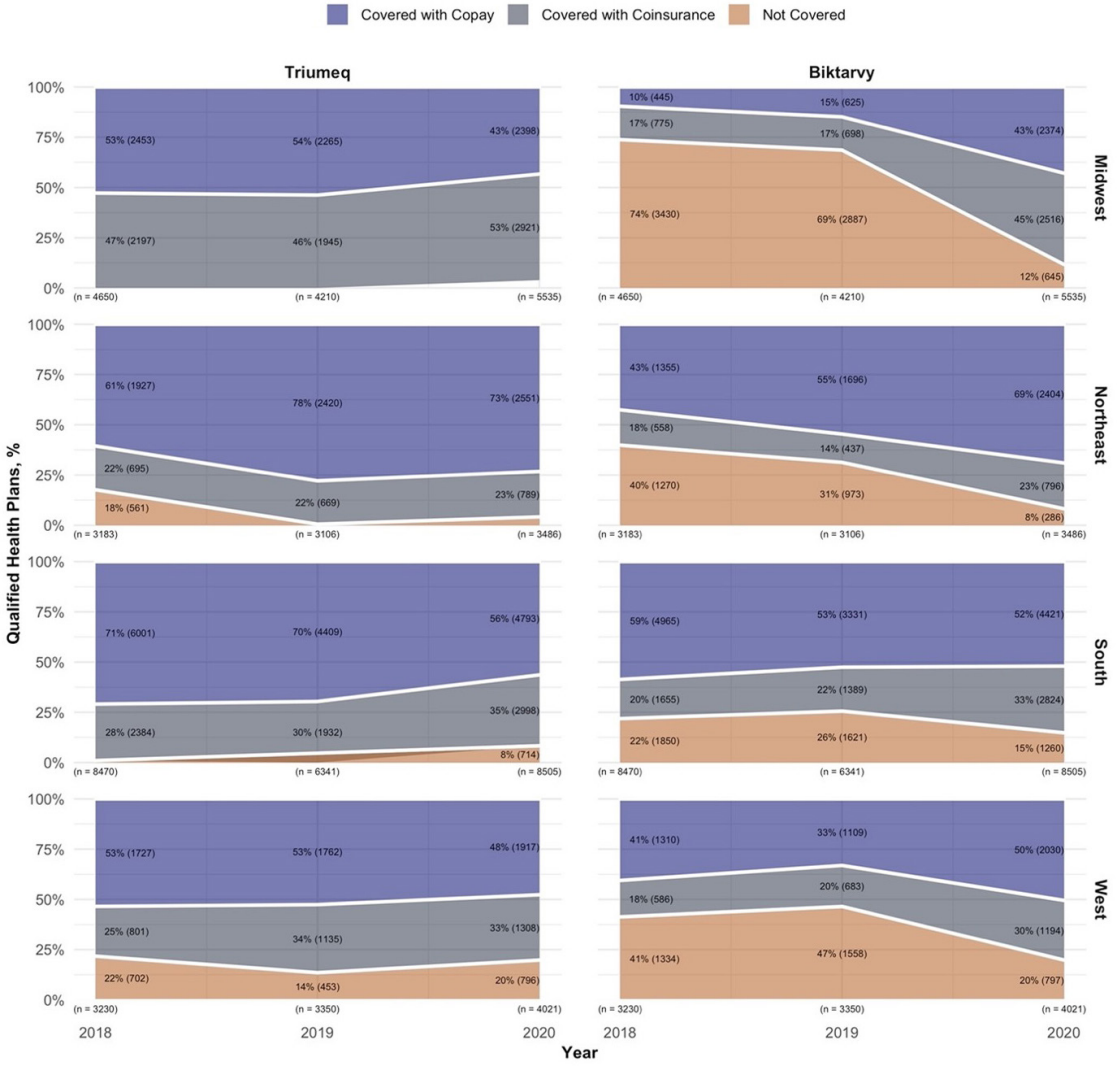

Figure 3. Qualified Health Plan Coverage and Cost Sharing for Triumeq and Biktarvy by “Ending the HIV Epidemic” Priority Jurisdiction Status, 2018-2020

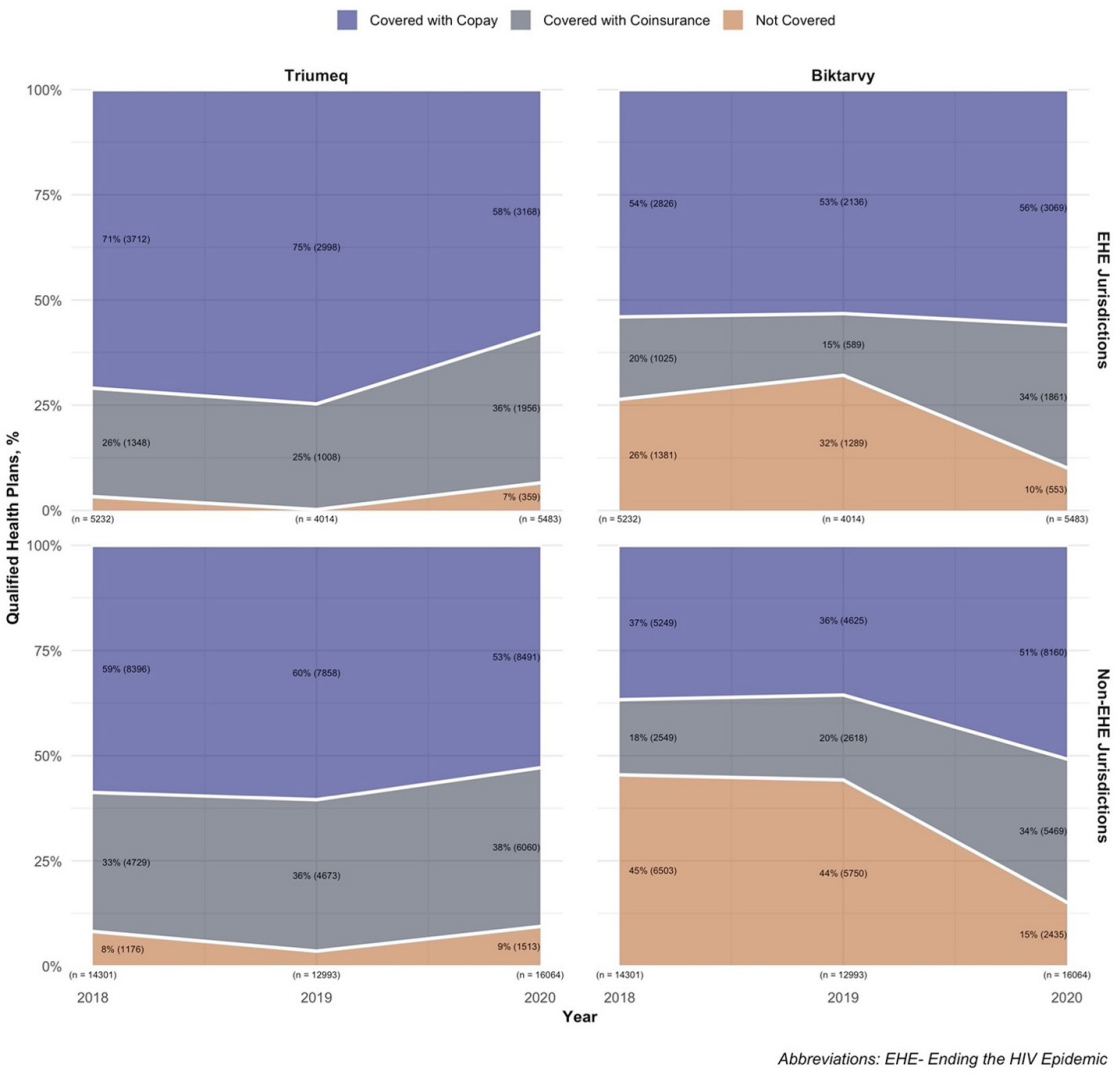

Figure 4. Monthly Out-of-Pocket Cost for Qualified Health Plan Premium and Triumeq or Biktarvy by Cost Sharing Type and (A) Region or (B) “Ending the HIV Epidemic” Priority Jurisdiction Status, 2018-2020

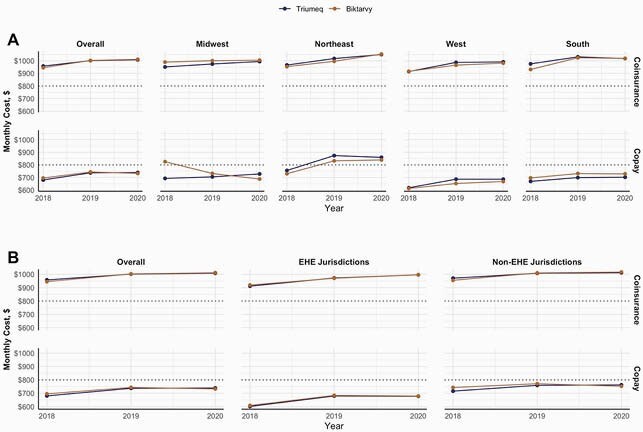

**Conclusion:**

STR coverage remains heterogenous across the United States. Over time, coverage of the newer STR increased, but many QHPs in EHE jurisdictions still required coinsurance. Access to newer ART regimens may be slowed by delayed QHP coverage or complex negotiations with manufacturers about formulary inclusion as ART options become more competitive, even if patients are insulated from cost differences.

**Disclosures:**

**Kathleen A. McManus, MD, MSCR**, **Gilead Sciences, Inc.** (Research Grant or Support, Shareholder)

